# Mitral stenosis and pregnancy: Current concepts in anaesthetic practice

**DOI:** 10.4103/0019-5049.71043

**Published:** 2010

**Authors:** M Kannan, G Vijayanand

**Affiliations:** Department of Anaesthesia and Critical Care, Tirunelveli Medical College, Under Government of Tamilnadu, Tamil Nadu - 627 011, India

**Keywords:** Labour analgesia and caesarean section, mitral stenosis, pregnancy

## Abstract

The incidence of rheumatic mitral stenosis is grossly reduced in India. Still, among heart disease complicating pregnancy, rheumatic mitral stenosis occupies a greater segment. The unique physiological changes in pregnancy and the pathological impact of mitral stenosis over pregnancy and labour are discussed in detail. A multidisciplinary approach in the diagnosis and management reduces the mortality and morbidity during peripartum. The labour analgesia technique and the evidence-based regional and general anaesthesia techniques are discussed at length in this article.

## INTRODUCTION

Rheumatic heart disease is still a major heart problem associated with pregnancy in India, despite its declining trend. The incidence of rheumatic mitral stenosis was 5.4 per 1,000 school children in 1995,[1] and it has been reduced to 0.5–0.64 per 1,000.[2,3] Rheumatic mitral stenosis forms 88% of the heart diseases complicating pregnancy in the tertiary referral centre in India.[4] The mortality and morbidity are considerably reduced[5] by better perinatal care, where the anaesthesiologist plays a major role in the multidisciplinary approach. Nevertheless, in the developed world, rheumatic disease has become uncommon and complex congenital heart disease is increasing in the recent decades. With the advent of intensive obstetric and anaesthetic care, the death rate of pregnant women with heart disease is lower in mitral stenosis compared with other congenital heart diseases like Eisenmenger’s syndrome, pulmonary vascular obstructive disease and Marfan’s syndrome with aortopathy.[6] Although mitral stenosis is often associated with mitral regurgitation, morbidity is usually related to mitral stenosis[7]

## PATHOPHYSIOLOGY OF MITRAL STENOSIS IN PREGNANCY

### Cardiovascular changes during pregnancy

Although the physiologic changes in the cardiovascular system appear to begin in the first trimester, these changes continue into the second and third trimesters, when the cardiac output increases by approximately 40% of the pre-pregnant values. The cardiac output increases from the fifth week of pregnancy and reaches its maximum levels by 32 weeks. Cardiovascular changes during pregnancy are summarized in Table 1. The cardiovascular changes of pregnancy resolve by 3–6 months after delivery. It may even take a year for the residual effects of cardiovascular remodeling to subside.

**Table 1:** Cardiovascular changes during pregnancy

Parameter	Percentage of change	
Cardiac output	40–50%	Increase
Stroke volume	30%	Increase
Heart rate	15–25%	Increase
Intravascular volume	45%	Increase
Systemic vascular resistance	20%	Decrease
Systolic BP		Minimal
Diastolic BP	20%	Decrease at mid-pregnancy Pre-pregnant values at term
CVP		Unchanged
O_2_ consumption	30–40%	Increase

When the normal mitral valve orifice area of 4–6 cm^2^ is progressively reduced to 2 cm^2^, the classical symptoms of mitral heart disease start appearing. Mitral stenosis prevents emptying of the left atrium and subsequent filling of the left ventricle, resulting in decreased stroke volume and decreased cardiac output. Consequent to the fixed cardiac output state, the heart cannot cope up with situations warranting increased metabolic demand or increased blood volume. When the stenosis progresses, the left atrium dilates and the left atrial pressure increases. A pressure gradient develops during diastole between the left atrium and the left ventricle. This pressure gradient is the haemodynamic hallmark of mitral stenosis. Hence, the back pressure on the pulmonary vessels leads to pulmonary congestion and, in severe cases, pulmonary oedema. Longstanding pulmonary venous congestion causes irreversible changes in the vessel wall, leading to chronic pulmonary hypertension.

Women with severe mitral stenosis often do not tolerate the cardiovascular demands of pregnancy. This increased volume load and tachycardia together cause the patients to deteriorate and advance from one New York Heart association (NYHA) class to another. The increased heart rate of pregnancy limits the time available for left ventricular filling, resulting in increased left atrial and pulmonary pressures and an increased likelihood of pulmonary oedema. When the pulmonary capillary pressure exceeds the blood oncotic pressure, pulmonary oedema develops. Atrial fibrillation worsens this scenario and about 80% of the cases of systemic emboli occur in patients with atrial fibrillation.

Cardiac decompensation and pulmonary oedema may occur in pregnant women with overt or silent mitral valve stenosis during the second or third trimester. The risk of maternal death is greatest during labour and during the immediate post-partum period. The sudden increase in the pre-load immediately after delivery, due to autotransfusion from the uterus, may flood the central circulation, resulting in severe pulmonary oedema. In addition, there continues to be autotransfusion of blood for 24–72 h after delivery. Thus, the risk of pulmonary oedema extends for several days after delivery.[8] The greatest risk occurs in the peripartum period, and most deaths occur between the second and ninth days post-partum. The haemodynamics during labour and puerperium are summarized in Tables 2 and 3.

**Table 2:** Haemodynamics during labour

Parameter	Stage of labour	Percentage of change	
Cardiac output	Latent phase	10%	Increase
	Active phase	25%	Increase
	Expulsive phase	40%	Increase
	Immediate post-partum	75–80%	Increase
Heart rate	All stages		Increase
CVP	All stages		Increase

**Table 3:** Haemodynamics during puerperium

Parameter	Post-partum	Percentage of change
Cardiac output	Within 1 h	30% above pre-labour values
	24–48 h	Just below pre-labour values
	2 weeks	10% above pre-pregnant values
	12-24 weeks	Baseline pre-pregnancy values
Heart rate	Immediate	Decrease
	2 weeks	Pre-pregnant values
Stroke volume	48 h	Remains above pre-labour values
	24 weeks	10% above pre-pregnant values

## DIAGNOSIS

Although clinical examination, electrocardiogram and X-ray chest reveal many relevant findings, echocardiography is the standard imaging tool used to assess patients with mitral stenosis. Echocardiography provides information regarding the area of the mitral valve, size of the left atrium, presence of thrombus and the size and function of the left ventricle and right-sided chambers. Doppler examination provides information about the severity of the stenosis, the presence of other associated valve lesions and the degree of pulmonary hypertension [Table 4]. Diagnostic cardiac catheterization is necessary only when echocardiography is non-diagnostic or results are discordant with clinical findings.

**Table 4:** Severity grading of mitral stenosis

Measurement	Normal	Mild	Moderate	Severe
Mitral valve area (cm^2^)	4.0–6.0	1.5–2.5	1.0–1.5	<1.0
Mean pressure gradient (mmHg)	<2	2–6	6–12	>12
Pulmonary artery mean pressure (mmHg)	10–20	<30	30–50	>50

### Prediction of mortality and morbidity

The maternal outcome seems to correlate well with the New York Heart Association (NYHA) functional classification.[9,10] Maternal cardiac complications, such as pulmonary oedema and arrhythmias, occurred in 35% of the pregnancies. The incidence of maternal cardiac complications correlates with the severity of the mitral stenosis (67% for severe, 38% for moderate and 26% for mild disease).[11] Mortality rates for class I and II amount to <1%, whereas they range between 5 and 15% for class III and IV. The peri-natal mortality rate for class III and IV is as high as 20–30%. Siu and others,[12] expanded on the NYHA classification and developed a system for predicting complications during pregnancy.

1. Prior heart failure, arrhythmia, transient ischaemic attack or stroke.
2. A baseline NYHA III class or more or cyanosis.
3. Systemic ventricular dysfunction (ejection fraction, 40%).
4. Pulmonary hypertension (pulmonary arterial systolic pressure 50% of systemic pressure).
5. Left heart obstruction.
  · Severe aortic stenosis (valve area, 1/cm^2^, Doppler jet velocity 4 m/s).
  · Symptomatic or severe mitral stenosis.
6. Severe aortic or mitral regurgitation with NYHA class III or IV symptoms.

### Management

Management of the pregnant woman requires a multidisciplinary team for optimal maternal and foetal outcomes. Antenatal management is directed towards avoiding cardiac decompensation, with regular assessment for volume overload and pulmonary oedema.

## MEDICAL MANAGEMENT

In symptomatic patients, medical treatment should be the first line of management. Treatment involves bed rest, oxygen therapy and diuretics. Beta-adrenergic receptor blockade is useful to prevent tachycardia during pregnancy. Propranolol or atenolol decreases the incidence of maternal pulmonary oedema without adverse effects on the foetus or neonate.[13] Recent trials conclude that digoxin has no role in prevention and in the treatment of cardiac failure.[14]

Atrial fibrillation requires aggressive treatment with digoxin and beta blockers to revert it to sinus rhythm and anticoagulation to prevent systemic embolization. Cardioversion should be performed if pharmacologic therapy fails to control the ventricular response. Anticoagulation, even in the absence of atrial fibrillation, is beneficial.[15,16] Because of the paucity of data regarding the efficacy of anticoagulants during pregnancy, recommendations concerning their use during pregnancy are based largely on extrapolations from the data from non-pregnant patients, from case reports and from case series of pregnant patients.[17] Due to the high incidence of embryopathy during the first trimester and bleeding during parturition, warfarin should be used during 12–36 weeks of pregnancy only. Standard therapy during pregnancy would be

- SC/IV heparin for up to 12 weeks antepartum (aPTT 1.5–2.5-times of normal)
- Warfarin from 12 to 36 weeks (maintain INR 2.5–3.0)
- SC/IV heparin after 36 weeks

Therapy with low-molecular weight heparin (LMWH) instead of unfractionated heparin is gaining popularity. Although an “anti Xa” activity is used to monitor LMWH, no anti-Xa activity-based guidelines have been issued till date.[18–20] Antibiotic prophylaxis for endocarditis is reserved only for patients with a previous history of endocarditis or presence of established infection.[21]

## SURGICAL MANAGEMENT

If mitral stenosis is diagnosed before pregnancy, mitral commissurotomy is preferred. During pregnancy, the second trimester is the preferred period for any invasive procedure. Percutaneous valvuloplasty using the Inoue balloon technique has become the accepted treatment for patients with severe symptomatic mitral stenosis. Percutaneous balloon mitral valvuloplasty provides palliation for pregnant women with mitral stenosis, and the reported success rate is nearly 100%. Successful balloon valvuloplasty increases the valve area to >1.5 cm^2^without a substantial increase in mitral regurgitation.[22] Although the maternal outcome in percutaneous balloon mitral valvuloplasty and open commissurotomy are the same, the foetal loss is high in open commissurotomy, at a ratio of 1:8.[23] Valve replacement is reserved for severe cases with calcified valve and in mural thrombus where the maternal mortality is 1.5–5% and the foetal loss is 16–33%.[24]

## OBSTETRIC MANAGEMENT AND LABOUR ANALGESIA

The role of the anaesthesiologist begins by providing good labour analgesia. Most reports have recommended vaginal delivery under epidural anaesthesia, unless obstetrically contraindicated. Caesarean section is indicated for obstetric reasons only. In a study by Goldszmidt and others,[25] only 29–31% of the 522 women with heart disease required caesarean section and nearly 70% of them underwent vaginal delivery under epidural analgesia. Tachycardia, secondary to labour pain, increases flow across the mitral valve, producing sudden rises in left atrial pressure, leading to acute pulmonary oedema. This tachycardia is averted by epidural analgesia without significantly altering the patient haemodynamics.[26] Invasive cardiac monitoring like radial artery cannulation and pulmonary catheter are beneficial in assessing the cardiac output, pulmonary artery pressure and for guiding fluid and drug therapy, especially in NYHA III and IV patients.[27,28] Sudden drops in systemic vascular resistance (SVR) in the presence of a fixed cardiac output can be prevented by small bolus doses of phenylephrine, with volume expansion when necessary.

Combined spinal–epidural analgesia during labour using intrathecal fentanyl 25 μg produces good analgesia without major haemodynamic changes during the first stage of labour. During the second stage of labour, only the uterine contractile force should be allowed rather than the maternal expulsive effort that is always associated with the valsalva maneuver. Therefore, the second stage of delivery should be cut short by instrumentation. Supplementary analgesia for instrumentation with slow epidural boluses of fentanyl and a low concentration of bupivacaine reduces SVR and the cardiac pre-load.[29] Low spinal anaesthesia for vaginal instrumental delivery has also been used with good results in these patients.[30]

Labour pain can affect multiple systems that determine the uteroplacental perfusion. Hence, foetal heart rate monitoring should be carried out during all stages of labour. Supplemental oxygen administration with pulse oximetry monitoring to minimize increases in pulmonary vascular resistance and maintenance of left uterine displacement for good venous return are mandatory. Supplementary epidural anaesthesia can be maintained throughout the immediate post-partum period and the catheter left *in situ* could provide anaesthesia for immediate or post-partum tubal sterilization.

### Anaesthetic management of abdominal delivery

The goals for the anaesthetic management of patients with mitral stenosis are: (1) maintenance of an acceptable slow heart rate, (2) immediate treatment of acute atrial fibrillation and reversion to sinus rhythm, (3) avoidance of aortocaval compression, (4) maintenance of adequate venous return, (5) maintenance of adequate SVR and (6) prevention of pain, hypoxaemia, hypercarbia and acidosis, which may increase pulmonary vascular resistance.

There are no controlled studies examining the best type of anaesthetic technique in these patients and guidelines and standards are lacking. Therefore, individualizing the anaesthetic management according to the parturient’s cardiovascular status and the practitioners’ knowledge and experience of the existing treatment options is the key to success in these patients.[29] For the past two decades, regional anaesthesia has proved to be a safe technique in cardiac patients presenting for caesarean section. Epidural and continuous spinal anaesthetic techniques are attractive options.

One of the major advantages of epidural analgesia is that it can be administered in incremental doses and that the total dose could be titrated to the desired sensory level. This, coupled with the slower onset of anaesthesia, allows the maternal cardiovascular system to compensate for the occurrence of sympathetic blockade, resulting in a lower risk of hypotension and decreased uteroplacental perfusion. Moreover, the segmental blockade spares the lower extremity “muscle pump,” aiding in venous return, and also decreases the incidence of thromboembolic events. Invasive haemodynamic monitoring, judicious intravenous administration of crystalloid and administration of small bolus doses of phenylephrine maintain maternal haemodynamic stability.[30]

Continuous spinal anaesthesia, although infrequently practiced, could be a better option in some rare situations like accidental dural puncture. Dresner conducted 34 caesarean sections with cardiac disease under continuous spinal anaesthesia with a success rate of 99%.[31] Neuraxial block in an anticoagulated patient has the risk of epidural haematoma. The Second ASRA Consensus Conference on Neuraxial Anesthesia and Anticoagulation (2003) has laid down valuable guidelines for such clinical situations.[32]

General anaesthesia has the disadvantage of increased pulmonary arterial pressure and tachycardia during laryngoscopy and tracheal intubation. Moreover, the adverse effects of positive-pressure ventilation on the venous return may ultimately lead to cardiac failure.[33]

Despite these disadvantages, if general anaesthesia is contemplated, tachycardia, inducing drugs like atropine, ketamine, pancuronium and meperidine, should be totally avoided. A beta-adrenergic receptor antagonist and an adequate dose of opioid like fentanyl should be administered before or during the induction of general anaesthesia. Because esmolol has a rapid onset and short duration of action, it is a better choice in controlling tachycardia. Since foetal bradycardia has been reported after esmolol, foetal heart rate should be monitored. Modified rapid sequence induction using etomidate, remifentanyl and succinylcholine is an ideal choice in tight stenosis with pulmonary hypertension.[34,35] Maintenance of anaesthesia can be carried out with oxygen and nitrous oxide 50:50, isoflurane, opioids and vecuronium. With associated severe pulmonary hypertension, nitrous oxide can be omitted. At this juncture, invasive haemodynamic monitoring is an inevitable guide.

After delivery of the foetus, oxytocin 10–20 u in 1,000 ml of crystalloid should be administered at a rate of 40–80 mu/min. An infusion of oxytocin can lower the SVR as well as elevate the pulmonary vascular resistance, resulting in a drop in cardiac output. Care must be taken during its administration. Methylergonovine, or 15-methylprostaglandin F_2α_, produces severe hypertension, tachycardia and increased pulmonary vascular resistance.[36,37]

Irrespective of the mode of delivery and anaesthetic technique, these patients are at a great risk of haemodynamic stress due to autotransfusion of blood from the uterus. This may lead on to pulmonary hypertension, pulmonary oedema and cardiac failure. Therefore, intensive monitoring and therapy should be continued till the haemodynamic parameters return to normal.

## CONCLUSION

Rheumatic mitral stenosis complicating pregnancy is still a frequent cause of maternal death. A better understanding of the physiological changes in pregnancy and the pathological impact of mitral stenosis over pregnancy and a multidisciplinary approach in diagnosis and management reduce the mortality and morbidity.
